# Stimulus information stored in lasting active and hidden network states is destroyed by network bursts

**DOI:** 10.3389/fnint.2015.00014

**Published:** 2015-02-23

**Authors:** Mark R. Dranias, M. Brandon Westover, Sidney Cash, Antonius M. J. VanDongen

**Affiliations:** ^1^VanDongen Laboratory, Program in Neuroscience and Behavioral Disorders, Duke-NUS Graduate Medical SchoolSingapore, Singapore; ^2^Massachusetts General HospitalBoston, MA, USA; ^3^Harvard Medical SchoolBoston, MA, USA

**Keywords:** optogenetics, stimulus memory, network excitability, interictal spike, epilepsy, transient cognitive impairment (TCI), Multielectrode array (MEA)

## Abstract

In both humans and animals brief synchronizing bursts of epileptiform activity known as interictal epileptiform discharges (IEDs) can, even in the absence of overt seizures, cause transient cognitive impairments (TCI) that include problems with perception or short-term memory. While no evidence from single units is available, it has been assumed that IEDs destroy information represented in neuronal networks. Cultured neuronal networks are a model for generic cortical microcircuits, and their spontaneous activity is characterized by the presence of synchronized network bursts (SNBs), which share a number of properties with IEDs, including the high degree of synchronization and their spontaneous occurrence in the absence of an external stimulus. As a model approach to understanding the processes underlying IEDs, optogenetic stimulation and multielectrode array (MEA) recordings of cultured neuronal networks were used to study whether stimulus information represented in these networks survives SNBs. When such networks are optically stimulated they encode and maintain stimulus information for as long as one second. Experiments involved recording the network response to a single stimulus and trials where two different stimuli were presented sequentially, akin to a paired pulse trial. We broke the sequential stimulus trials into encoding, delay and readout phases and found that regardless of which phase the SNB occurs, stimulus-specific information was impaired. SNBs were observed to increase the mean network firing rate, but this did not translate monotonically into increases in network entropy. It was found that the more excitable a network, the more stereotyped its response was during a network burst. These measurements speak to whether SNBs are capable of transmitting information in addition to blocking it. These results are consistent with previous reports and provide baseline predictions concerning the neural mechanisms by which IEDs might cause TCI.

## Introduction

Cellular and network memory mechanisms underlie psychologically relevant processes like working memory and perception. These basic memory mechanisms include “hidden” and “active” mechanisms which reference the short-term adaptation of neurons to repeated stimulation as is revealed in “paired-pulse” experiments and the remnants of stimuli that persist as reverberations of action potentials in neuronal circuits (Buonomano and Merzenich, 1996; Mongillo et al., 2008; Buonomano and Maass, 2009). In epilepsy, the performance of many tasks that rely on these basic memory mechanisms, from motor planning to perception to working memory, can be disrupted by abnormal focal discharges of synchronized neural activity between seizures, an effect known as transitory cognitive impairment or TCI (Binnie et al., 1987; Stafstrom, 2010). These abnormal discharges last 70–200 ms and are known as interictal epileptiform discharges (IEDs; de Curtis and Avanzini, 2001; Binnie, 2003). IEDs likely arise from excessively synchronous inputs to a focal set of neurons that are possibly impaired by ion channel abnormalities or activated by the local release of glutamate by glia (Rogawski, 2006). Recently, a rodent model of TCI was developed using the short-term memory task, delayed match to sample (DMS; Kleen et al., 2010). The DMS task has three phases: an encoding phase where the first stimulus (the “sample”) is presented, an intervening delay phase, and a recall phase where matching and mismatching cues are presented to elicit responses. Kleen et al. (2010) recorded hippocampal IEDs throughout the DMS task, but found only hippocampal IEDs occurring during the recall phase of DMS impaired performance. The authors argue this indicates the hippocampus only processes DMS-relevant information during the recall phase. However, the depth electrodes used in these experiments could not resolve whether the activity of hippocampal neurons encodes stimulus-specific information nor could they show whether this information was maintained or destroyed by IEDs. The current study uses multielectrode array (MEA) recording and optogenetic stimulation to investigate whether neuronal networks continue to represent stimulus-specific information after synchronized bursts of network activity have occurred. Using a laser projection system, optogenetically modified, dissociated cultures of cortical neurons can be optically stimulated with complex stimuli such as random dot patterns (Dranias et al., 2013). When these neurons are plated on MEAs, the network activity that results from stimulation can be recorded and the firing rate of neurons and patterns of recruitment encode the identity of stimuli for hundreds of milliseconds after the stimulation has been removed (Dranias et al., 2013). In addition to displaying the ability to encode stimulus information in neuronal firing rates, cultured neuronal networks can maintain stimulus-specific information across delays where no network activity has been observed for hundreds of milliseconds (Buonomano and Merzenich, 1996; Dranias et al., 2013). In these cases, stimulus information is said to be represented by hidden memory mechanisms and can be revealed using protocols like paired-pulse facilitation which are sensitive to synaptic adaptation and involve the sequential presentation of stimuli across a delay (Buonomano and Maass, 2009). A number of theorists and computational modelers have posited that this simple mechanism of stimulus-specific adaptation is the primary mechanism the brain relies on when performing novelty and familiarity detection in DMS-like tasks (Grossberg, 1980; Brown and Xiang, 1998; Brown and Aggleton, 2001; Yassa and Stark, 2008).

Synchronous Network Bursts (SNBs) arise spontaneously in cultures of living neuronal networks and appear to be an intrinsic property of any densely connected recurrent neural network (Wagenaar et al., 2005; Chiappalone et al., 2009; Hales et al., 2012; Maheswaranathan et al., 2012). Given that cultured neuronal networks can maintain stimulus-specific information across short delays, two experiments were performed to test whether this information is disrupted by SNBs. In the first experiment one of four possible stimuli was presented on each trial and trials interrupted by SNBs were compared to control trials to measure how much stimulus information was lost. A multiclass (4 class) Support Vector Machine (SVM) classifier is used to analyze these trials. In the second experiment a sequence of two stimuli is presented separated by a short delay. A binary (2 class) SVM classifier is used to analyze these trials. Like the paired pulse experiment, this experiment aims to measure whether information about prior stimulation is stored across a delay where there is no neural activity. Unlike the paired pulse experiment, the sequential stimulus experiment aims to detect evidence of stimulus-specific information, not just evidence of prior stimulation. In the sequential stimulus protocol, the identity of the first stimulus varies while the identity of the second stimulus is fixed. The adapted response of the network to the second stimulus is analyzed to measure how much information it contains about the first stimulus. In order to test whether stimulus-specific information survives an SNB, experiments were broken into three phases: encoding (first stimulus), delay, and recall (second stimulus). Once it was established that SNBs destroy stimulus-specific information, the firing rate, entropy, and similarity of network responses during SNBs were measured. It was hypothesized that if the SNBs act as white noise and interfere with the stimulus representation, network response patterns should be dissimilar and these trials will have a high entropy. As an alternative it was hypothesized if SNBs ‘overwrite’ stimulus-elicited responses by saturating active units then SNB network response patterns should be similar and have a low entropy.

## Materials and methods

### Primary neuron cell culture

E18 Sprague-Dawley rat pups are decapitated and utilizing aseptic technique, cortical tissue is dissected from the embryonic brain and placed directly into a 15 ml sterile plastic vial containing 10 ml ice-cold HBSS or Hibernate-E medium (BrainBits)^1^ and brought to a laminar flow hood for extraction of neurons from the cortical tissue. E1 is defined as the day after the plug is determined to be sperm-positive (Poon et al., 2014). All procedures carried out were approved by the Institutional Animal Care and Use Committee (IACUC) of the National University of Singapore. Poly-D-lysine and fibronectin coated 60 electrode MEA-containing culture dishes (Multi Channel Systems) are prepared as described previously (Van de Ven et al., 2005; Dranias et al., 2013). Cortical neurons from multiple pups are dissociated, and plated onto MEAs in aliquots of 40 uL at a density of 1 × 10^5^ neurons per MEA dish. Prior to plating, neurons are transfected with plasmid DNA encoding ChannelRhodopsin-2 (ChR2, a kind gift from Karl Deisseroth) fused to EYFP for visualization and carrying mutations H134R and T159C which were introduced to increase current (Nagel et al., 2005). Transfection was carried out using electroporation (Amaxa nucleofector II device and kit, Lonza Inc.) After electroporation and plating, MEAs were filled with approximately 1 mL NB-Active 4 cell medium (BrainBits) with 10% fetal bovine serum (FBS), covered with a plastic cap with Teflon film (ALA-Scientific), and the dish was placed into the incubator (37C, 5% CO_2_). The cell medium was replaced every 2–5 days and Yellow Fluorescent Protein (YFP) expression was visible within 24 h of transfection.

### MEA recordings

Extracellular electrophysiological recordings of neurons were made from 60 electrode MEA dishes using the MEA1060 hardware system (Multi Channel Systems). Recordings were performed on an anti-vibration table and in a Faraday cage. During experimental recordings, the cell culture medium (NBActive4) was replaced with Dulbecco’s phosphate-buffered saline containing glucose and pyruvate (DPBS, Sigma). MC_Rack software (Multichannel Systems) was used to acquire extracellular signals that were high pass filtered at 300 Hz and low pass filtered at 3 kHz with 2nd order Butterworth filters. Action potentials or “spikes” were detected using a voltage threshold rule. The value of the threshold was between 7–12 μV and was determined by the user for each dish based on the observed amount of channel noise. Electrophysiological data was imported into MATLAB using the Neuroshare API library.^2^

### Optical stimulus presentation and imaging

The MEA system was mounted on an inverted microscope during recordings (Eclipse Ti, Nikon). Fluorescent and Brightfield images were captured from the MEA dishes via a cooled CCD camera (Orca, Hamamatsu). Optical stimuli were presented onto the MEA using a 25 mW 488 nm laser (Spectra-Physics) beam which was passed through an acousto-optic tunable filter (AOTF, AA Opto-Electronic), optically expanded, passed through a polarizing filter and projected onto a reflective LCoS Spatial Light Modulator microdisplay (SLM, Holoeye Photonics AG) (Dranias et al., 2013). Blue light patterns reflecting off the SLM were passed through a second polarizing filter and projected onto the neuronal network growing on top of the MEA. All elements of the optical projection system were bolted to the anti-vibration table. TTL pulses generated by MATLAB synchronize recordings and stimulus presentations. The random dot stimuli were constructed from 18–22 randomly positioned squares on a 10 × 10 grid and had an image size of approximately 1.25 mm square when projected onto the MEA dish with an effective light intensity of 0.1 mW/mm^2^.

Beginning at 5 days *in vitro* (DIV), cultures were screened for ChR2-YFP expression. Cultures exhibiting YFP expression in the range of 1% +/− 0.5% were monitored for spontaneous single unit electrophysiological activity. Optical stimuli of increasing spatial resolution were presented to active dishes to test for functional expression of ChR2: networks showing a differentiated response to squares in different locations of a 2 × 2 grid were then tested with patterns of random dots from a 10 × 10 grid. Dishes showing a differentiated response to at least 5 of 30 random dot patterns were selected to undergo further study. In addition, networks in this study needed to have a limited but useful number of SNBs. Each step in this screening process eliminates about $\frac{1}{2}$ of dishes. Data arises from separate batches: 1905- Dish 1, Dish 4; 0504- Dish3; 2106- Dish 3, Dish 5.

### Experimental protocols

Random dot stimuli consisted of 18–22 randomly positioned squares on a 10 × 10 grid occupying 1.25 mm^2^ on an MEA dish. Single stimulus presentation experiments are used to test whether SNBs disrupt stimulus information represented in lasting network activity. During single stimulus presentations one of four random dot stimuli is presented for 100–200 ms. A multiclass (4 class) SVM classifier was used to analyze these trials to identify stimulus-specific information (see section Stimulus Information Time Series). Sequential stimulus presentation experiments are similar to paired-pulse experiments and aim to test whether SNBs disrupt hidden network representations of stimuli. During sequential stimulus presentations the first stimulus (cue) is presented for 100–200 ms, followed by a delay period of 1 s after which the second probe stimulus is presented. While cue stimuli vary on different trials, the probe stimulus is the same on every trial. Two cue stimuli were alternated on trials so a binary (2 class) SVM classifier is used to analyze these trials (see section Stimulus Information Time Series ). Responses to the probe are analyzed to see if they reflect information about specific cue stimuli. Like paired pulse experiments, the sequential stimulus experiments are used to detect evidence that the network stores information in the absence of neural activity. However in the sequential stimulus task the stimuli differ and the information to be measured regards the identity of past stimuli, rather than simple evidence of past stimulation. In order to minimize the possibility that action potentials are transmitting stimulus information during the delay period, unit activity is monitored during sequential stimulus trials and trials with unit activity during the final 200 ms of the delay period are flagged for later analysis. The persistence of cue-specific information was measured in both trials using a time-series constructed from Support Vector Machines (SVMs; see below).

### Experimental trials with and without SNBs

Network responses were sorted into trials with and without SNBs. During single stimulus presentation trials, SNBs were detected using a threshold rule of more than 20 spikes in the first 590 ms. During sequential stimulus presentation experiments “control trials” are those trials where no SNBs occur until after the second (probe) stimulus. This protocol aims to investigate information stored using hidden mechanisms so control trials are additionally restricted to trials where there is no unit activity during the final 200 ms of the delay period. Trials with SNBs were divided into three types based on the phase in which an SNB occurred: cue, delay, or probe. A cue phase trial with SNBs was deemed to occur if an SNB occurred prior to or coincident with the cue stimulus. A cue period SNB was identified whenever half the mean number of spikes per trial occurred in the first 590 ms of the trial. A delay phase trial with SNBs was deemed to occur when an SNB was observed between cue and probe stimuli. The delay phase SNB was identified whenever half the mean number of spikes per trial occurred in the interval between cue and probe, followed by a 100–300 ms pause in which no spikes were observed prior to presentation of the probe stimulus. Probe phase trials with SNBs were deemed to occur whenever an SNB immediately preceded or coincided with the probe stimulus. The probe SNB was identified when at least 20 spikes occurred in a 300 ms time window starting from 100 ms prior to probe presentation until 100 ms after probe presentation. Trials presented in figures were selected in order to convey the typical network responses and do not represent observed frequencies of each trial type; rather trials are typically presented in some equally weighted distribution of across classes (50–50 or 33-33-33).

### Stimulus information time series

Support vector machines (SVMs) were used to distinguish network responses to different stimuli.

The SVM time series is constructed using multiple, independent SVMs to measure how stimulus information varies over time (Nikolić et al., 2009; Dranias et al., 2013). Each SVM analyzed a 100 ms time bin and is trained to recognize differences in the pattern of recruitment and firing rate of neurons in that time window. SVM classifiers label network responses on single trials according to the stimulus it predicts was presented. SVMs perform either 4-choice classifications (single stimulus task) or 2-choice classifications (sequential stimulus task) and are implemented in MATLAB using *libsvm* (Chang and Lin, 2011). The baseline or chance rate of classification was either $\frac{1}{4}$ or $\frac{1}{2}$, depending on the number of stimuli used in the experiment as all stimuli were presented an equal number of times (in blocks of 64 pseudorandom trials).

Data points making up the stimulus information time series were computed by SVMs focused on classifying data from a single time bin. Using notation, the construction of the SVM array and time series can be understood more precisely. Each trial was divided into *n* 100 ms bins:

$$\left(bin_{1},bin_{2},bin_{3},\ldots bin_{n}\right)$$

Hence for a 2 s trial, there would be 20–100 ms time bins (*n* = 20). An independent SVM classifer is assigned to analyze data in each time bin:

$$\left({\text{SVM}}_{\text{1}}\text{,}{\text{SVM}}_{\text{2}}\text{,}{\text{SVM}}_{\text{3}}\text{,}\ldots {\text{SVM}}_{n}\right)$$

In the case of a 2 s trial (*n* = 20), there would be 20 independently trained SVMs, each focused on analyzing the data from a corresponding time bin. Data in every time bin was constructed by computing a population spike count vector. Each spike count vector, *spike*_i_ (where *i* corresponds to *bin*_i_), is 60 dimensional (59 electrodes and a ground) and records the number of spikes seen in each unit in a 100 ms time bin. The 60th channel (ground) was assigned a default value of 1 in every time bin (preventing dividing by zero). Hence each vector is:

$$spike_{i}={\text{(count}}_{\text{1}}\text{,}{\text{count}}_{\text{2}}\text{,}{\text{count}}_{\text{3}}\text{,}\ldots {\text{count}}_{\text{60}}{\text{)=(count}}_{\text{1}}\text{,}{\text{count}}_{\text{2}}\text{,}{\text{count}}_{\text{3}}\text{,}\ldots {\text{count}}_{\text{59,}}\text{1)}$$

Hence on a given 2 s trial, *j*, there would be 20 spike vectors, corresponding to each time bin:

$$spike_{1,j},spike_{2,j},spike_{3,j},\ldots spike_{20,j}$$

Each SVM classifier is focused on analyzing the data of a single time bin and uses multiple trials worth of spiking data during training and testing. Typically 70% of the trials for a given experiment were used for training an individual SVM and the remaining 30% of trials for testing. Hence for an experiment where there are 800 trials, SVM_*7*_ in *bin_7_*, would be trained on the set of spike data:

$$\left\{spike_{7,1},spike_{7,2},spike_{7,3},\ldots spike_{7,560}\right\}$$

But then the SVM_7_ model is tested on the remaining spike data:

$$\left\{spike_{7,561},spike_{7,562},spike_{7,563},\ldots spike_{7,800}\right\}$$

The average accuracy across all training or testing trials is then reported. Only data that is linearly separable will have an accuracy of 100%. The stimulus information time series is contructed by presenting the average accuracy of individual SVMs as time-ordered data points. To control against bias on individual training or testing sets, each SVM retrained and tested 50 times using different subsets of spike count data and the mean accuracy across these 50 training and testing epochs is reported in the stimulus information time series. In figures a red line typically indicates the amount of stimulus information during the training phase and a blue line indicates the amount of stimulus information during the testing phase.

When measuring how much information was destroyed on trials where an SNB occurred, the SVM When measuring how much information was destroyed on trials where an was trained on trials where no SNB occurred and then tested on trials with SNBs. Methods for quantifying the accuracy, significance, reliability, and generalization of classifier results are discussed in statistical methods.

### Binary network activity vectors

Patterns of network activity were reduced to a binary vector that indicated whether a given channel was active or not in a 250 ms time bin. A unit is active when its firing rate is 3 STD above its inter-trial interval firing rate, similar to the rule for characterizing neuronal avalanches (Beggs and Plenz, 2004; Pasquale et al., 2008; Chen et al., 2010). Time bins were fixed at 250 ms windows to facilitate averaging and comparisons across different trials. The duration of the window was selected because it captures the initial stimulus-elicited network response, separating it from the subsequent network bursting response. The ground electrode channel was assigned a value of one rather than zero, preventing undefined division operations.

### Entropy time series

In order to measure the number of different ways the network responds to stimuli, a time series was constructed by breaking the data into 250 ms time bins and counting the number of different binary network activity vectors observed across all trials. Some binary network activity vectors occur more frequently than others and in order to measure this stereotypy, the number of exemplars of each binary network activity vector is counted and these tallies are used to compute the entropy. Smaller entropy values indicate network responses during a given time bin are highly stereotyped while higher entropies during a given time bin indicate the patterns of network responses are diverse, with the upper limit of different response patterns being the number of observed trials. Entropy is computed by counting the number of unique binary network activity vectors that occurred in each time bin and then adjusting this number by the frequency that each unique binary network response occurred:

$$H\left(x\right)=\log_{2}N-\frac{1}{N}\sum_{i}n_{i}\log_{2}n_{i}$$

Where “*x*” represents the outcome space of observed network responses, *H* is the entropy, *N* is the total number of binary network activity vectors, and *n_i_* represents the count of binary network activity vectors in each class, *i*, of equivalent binary vectors. To make the entropy an intuitive measure of how stereotyped the network responses are, entropy is plotted as the “equivalent number” of distinct network responses that would be associated with a given entropy value under the assumption that network responses arise from a uniform distribution. Hence, for each time bin, the entropy is plotted as 2H(x), giving the equivalent number of outcomes when the outcome space is composed of equally weighted classes. Time bins in the time series were set to 250 ms, except for the first time bin which was 550 ms. A weakness with entropy measurements is that they count the number of different responses but not how different the response are from each other.

### Cross-correlation matrix of binary network activity vectors

Data was broken into 250 ms time bins and a cross-correlation matrix was computed to compare the binary network activity vectors recorded on different trials. The cross correlation matrix was computed using the module *clusterdata* from the Statistics Toolbox in MATLAB. After the cross correlation matrix was computed, trials were sorted into clusters using a dendrogram algorithm that clusters similar network responses. After the network responses had been clustered by similarity, the trials in each cluster were examined to see which stimulus had been presented and the trials within the cluster were re-sorted by stimulus identity. Using this clustering approach it is possible to see whether two different neural responses are similar to each other despite having different binary network activity vectors (Raichman and Ben-Jacob, 2008). Hence the cross-correlation matrix complements entropy measurements by showing whether different network responses can be clustered into similar responses; this can indicate that some of the trial-to-trial variations in network responses are due to noise rather than fundamentally different patterns of activity. In diagrams, clusters of similar responses form reddish squares along the diagonal.

### Statistical methods

Machine classifiers known as SVMs were used to analyze single trial network activity and predict the identity of the stimulus driving that activity. Several approaches were used to quantify the accuracy, significance, specificity, reliability, and generalization of the classifiers. The accuracy of the classifier on both training and testing sets is reported using the mean correct classification rate, which is the complement of the misclassification rate (which is sometimes characterized using loss functions). The significance and specificity of SVM classification on single trials is established using 200 trials of random label shuffling, a monte carlo approach to characterizing how the classifier treats a randomly labeled data. The best and worst classification accuracy rates (5th and 95th percentiles) were recorded each classifier as dotted lines about the baseline (theoretical) chance rate of correct classification (either $\frac{1}{4}$ or $\frac{1}{2}$, depending on the number of stimuli). These confidence intervals help establish the significance of the correct classification rates. To insure generality and reliability of classifier results, single trials of network responses from each experiment were broken into training (70%) and testing sets (30%) for cross validation with repeated random subsampling. The repeated random subsampling controls for how bias relating to the unfair sampling of training and testing vectors affects classifier performance. Classifiers were retrained and tested 50 times and for each repeat, a different set of training and testing data is randomly selected from the experimental data. The average accuracy of the classifier on both training and testing sets is reported with the standard error of the mean (SEM). The mean (correct) classification rate and SEM demonstrate the reliability and generality of classifier methods (similar to the misclassification rate (the complement of accuracy) or a derivative loss function). In classification figures, the average classification accuracies are reported with solid lines and standard errors with gray shadows. When comparing trials with and without SNBs, modulations of accuracy in classification are recorded as mean percent of the values without SNBs, with standard error. The entropy of two categories of trials is compared: trials with and without SNBs. However, the number of trials in each category is not equal. In order to directly compare the entropy of these two categories, a random sample of trials is taken from the larger category, equal in size to the number of trials in the smaller category. This random sampling is repeated 300 times and the mean entropy is reported along with the 99th percentile extremes of the mean values seen across the resampling process are plotted as gray shadows behind the mean trend line wherever entropy was reported. For the category with the smaller number of trials, the entropy is computed directly. Wherever variables such as firing rate, number of active channels, and normalized entropy are compared across different networks, these variables were first standardized within each network. Standardization was done by computing the means and variances for each variable across all time bins. The values for firing rate, channel number, or entropy were then replaced with a standard score in each time bin and correlations between variables over time were computed after pooling data across all networks (or over a specified local time range):

$$r=\frac{1}{nm-1}\sum_{ij}\left(\frac{X_{ij}-M_{X}}{S_{x}}\right)\left(\frac{Y_{ij}-M_{Y}}{S_{y}}\right)$$

Where *M_X_* and *M_Y_* represent the mean values of the network-specific standardized variables *X_ij_*, *Y_ij_* pooled over all networks *j* and time bins *i*.

## Results

### Synchronizing network bursts (SNBs) disrupt network responses to single stimuli

**Figure 1:**
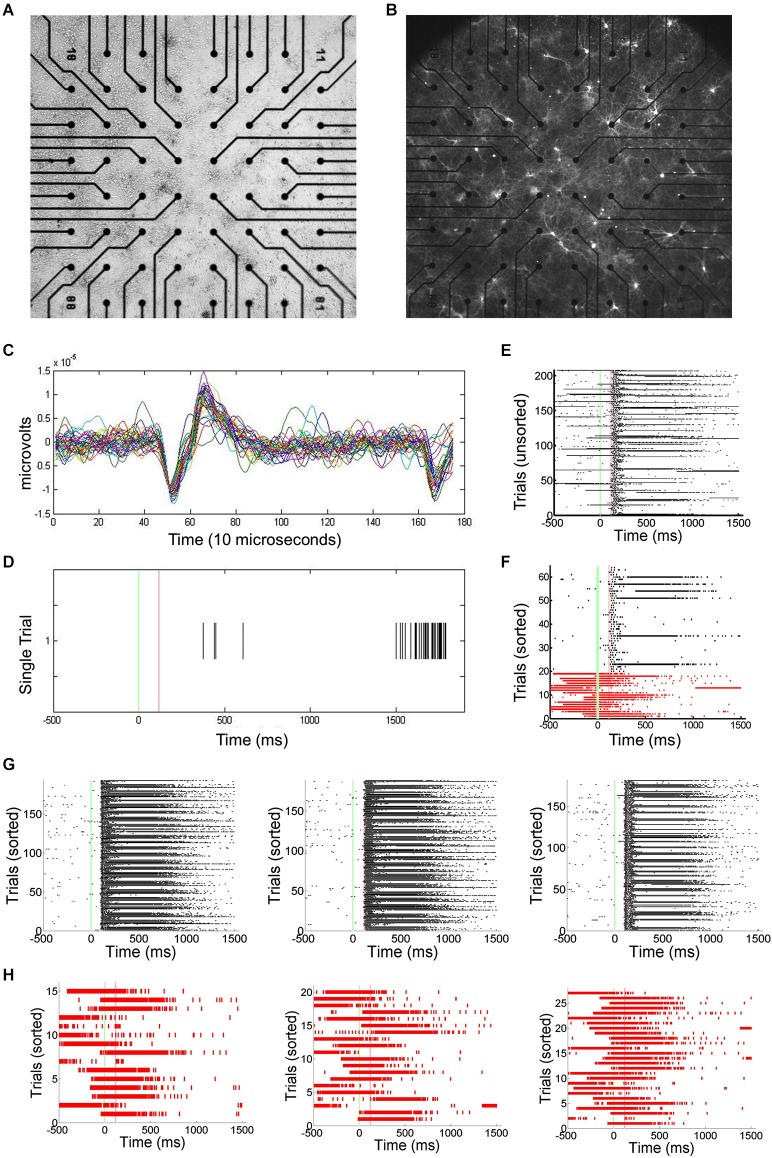
**Primary Culture, ChR2 transfection, and Multielectrode Array (MEA) Signals. (A)** Light microscopic image of primary neuronal culture at 8 days *in vitro* (DIV9) on a 60 electrode microelectrode array (MEA) transfected with Channel Rhodopsin-2 (ChR2) plasmid DNA coupled to Yellow Fluorescent Protein (YFP). **(B)** Image taken using a 4X objective and 510 nm excitation light to visualize ChR2-YFP expression. 2405-Dish3. **(C)** The MEA samples unit activity at 22 kHz. A threshold for detecting spikes in voltage is set based on observation of background noise levels. **(D)** When a threshold depolarization event or spike is detected, the “spike time” is recorded along with a 3ms clip of the waveform and saved in a data file. **(E)** Peristimulus rasterplots of spike times. Spike times are pooled from across all units in the network. TTL trigger signals are recorded and used to align data to stimulus onset, allowing the raster plots to be created. Each row indicates one trial. No consistent trends in SNB frequency across time were observed. Time from stimulus presentation shown on *x*-axis (ms), trial number on *y*-axis. Data from response to “stimulus 2” by 1905-Dish4. **(F)** Recorded trials sorted according to the whether or not a spontaneous network burst (SNB) interrupts the presentation of an optical stimulus and analyzed. Other conventions as **(E)**. **(G)** Peristimulus raster plots showing spiking responses of network, pooled across all units. Each row indicates one stimulus presentation. Stimulus identity varies from left to right: on left, responses to stimulus 1; center, responses to stimulus 3; on right, responses to stimulus 4. Data from trials without SNBs. Other conventions as **(E)**. **(H)** Peristimulus raster plots showing spiking responses of the network pooled across all units on trials with SNBs. Stimulus identity varies from left to right: on left, responses to stimulus 1; center, responses to stimulus 3; on right, responses to stimulus 4. Other Conventions as **(G)**.

Primary cortical neurons were cultured and transfected using ChR2 (Figures 1A,B). Static images of random dots were optically projected onto the networks, eliciting responses typically lasting 100–200 ms. Signals associated with this stimulation were electrophysiologically recorded using an MEA and spikes recorded by each electrode are translated into spike times (Figures 1C,D). Approximately 1–5% of trials were interrupted by SNBs. The occurrence of SNBs appeared unchanged across the recording session of single stimulus presentations (unsorted data shown in Figure 1E, sorted shown in Figure 1F). Simple stimulus presentation experiments involve presenting one of four stimuli to the dish in a pseudo random order. Figures 1E–H shows the responses of one network to four different stimuli (Batch 1905-Dish 4).

**Figure 2:**
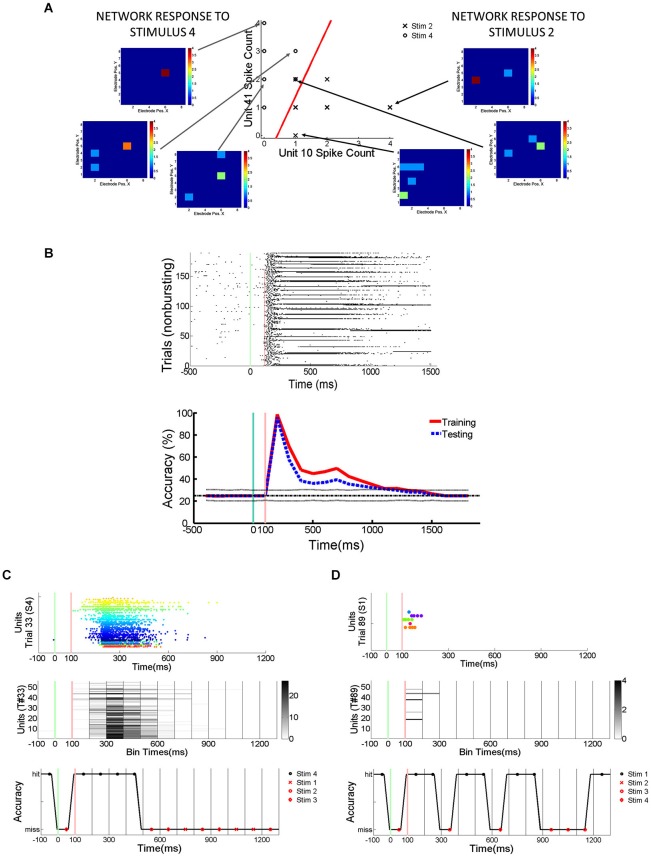
**Detection of Stimulus Information Using Linear Support Vector Machines (SVMs). (A)** Network responses to 12 stimulus presentations (data from 2106-Dish 5, DIV11). Six trials involve the presentation of stimulus 2 and 6 trials stimulus 4. On left and right are examples of network responses represented as heatmaps. *Left*: heatmaps showing network responses on three different presentations of Stimulus 4. Network represented by 8 × 8 colored arrays in which each colored cell represents an electrode position and the cell color represents spike rate (color code ranges from blue to red indicating 0 to 4 spikes per 100 ms). *Right*: heatmaps showing network responses to three presentations of Stimulus 2. Same conventions and color codes described for stimulus 4 (left). *Center*: Scatter plot showing responses of two units across these 12 representative trials. Responses to stimulus 2 are indicated by an “X” and responses to stimulus 4 are indicated by an “O”. Data points reflect the number of spikes observed at each unit in a 100 ms bin starting 200 ms post-stimulus. The *x*-axis indicates the number of spikes recorded from the unit at electrode 10 while the *y*-axis counts the number of spikes from the unit recorded at electrode 41. The line in red is the projection of the decision boundary used by the SVM to classify stimulus 2 from stimulus 4. Classification is effected by taking the inner product of the decision boundary vector with the spike count vector of an individual trial. Inner products with positive values are assigned to class 1 and negative values to class 2. The scatter plot is a restricted view of the overall network activity and examples of network-wide activity shown on left and right reveal additional units may be active on every trial. **(B)** Spikes recorded from cultured neuronal networks are counted and classified using an array of linear SVMs. *Top*: Peristimulus raster plot of network spikes (pooled across all units) to stimulus 2 on trials at are not interrupted by SNBs. Other conventions as Figure 1E. *Bottom*: Average accuracy of SVM classification across training trials (solid red line) and testing trials (solid blue line). A unique linear SVM is assigned to every 100 ms bin and each SVM is trained to classify only data from that time bin. SVMs are trained using a “batch mode” algorithm. 70% of single trial data is used for training and 30% of single trial data is used for testing classifier generalization. Red line plots the average accuracy with which a linear SVM can classify trials from the dataset it was trained on. Accuracy below 100% indicates that network responses in the training set are not linearly separable. The chance rate of classification is 25% for experiments where four stimuli are presented (solid black line). Dotted lines about the chance level (black line) represent the highest (90th percentile) and lowest (10th percentile) rates of accurate classification seen after 200 Monte Carlo simulations using a trained SVM but with datapoints that are randomly assigned to different classes (random relabeling). The blue line represents the mean classification accuracy observed when trained SVMs classify data from testing trials. Time is represented on *x*-axis, classification accuracy on *y*-axis (percentage of single trials classified correctly). **(C)** Application of trained SVM model to a single trial of test data (trial #33, stimulus 4, Figure 1G). *Top*: Peristimulus rasterplot of all units recorded from network on a single trial. Each unit is shown on a single row, spikes are shown as colored dots, the color of the dot is specific to the unit, aiding discrimination of which spike belongs to which row (unit). *y*-axis indicates unit number, *x*-axis time (ms). *Middle*: Peristimulus graph of the spike count associated with each 100 ms bin. The spike count in each 100 ms bin is encoded by intensity (colorbar at right provides a key for interpreting spikes counts). Time relative to stimulus onset shown on *x*-axis (ms), *y*-axis encodes units. *Bottom*: Accuracy of individual SVMs associated with each 100 ms time bin. Each SVM is either correct or incorrect (*y*-axis indicates “hit” or “miss”). Correctly classified time bins are indicated by a black marker, incorrectly classified time bins are indicated by a red marker. *x*-axis indicates time in ms, with a different SVM assigned to analyze data from every 100 ms time bin. **(D)** Analysis of spiking activity from a different single testing trial (trial #89, stimulus 1, Figure 1G). Other conventions as **(C)**. Data in **(B–D)** from 1905-Dish4.

In order to quantify how much stimulus information is lost during trials with SNBs, SVMs were trained to classify the electrophysiological responses of neuronal networks to different random dot stimuli. SVMs are linear classifiers and they classify data by separating them with linear decision boundaries (Figure 2A). The SVMs were trained using 70% of the trials without SNBs (training set; Figure 2B). The array of SVMs is unable to classify neural responses at an accuracy of 100%, even on its training set (classification accuracy on training set is indicated by a red line in the graph at bottom of Figure 2B). This indicates that network responses to different stimuli are not linearly separable. Figures 2C,D provide examples of how the array of classifiers (optimized using training data) analyze single trials from the remaining 30% of trials (the “testing set”). Overall generalization was good and the classification accuracy for testing data was comparable to training data (blue dashed line, graph at bottom of Figure 2B).

**Figure 3:**
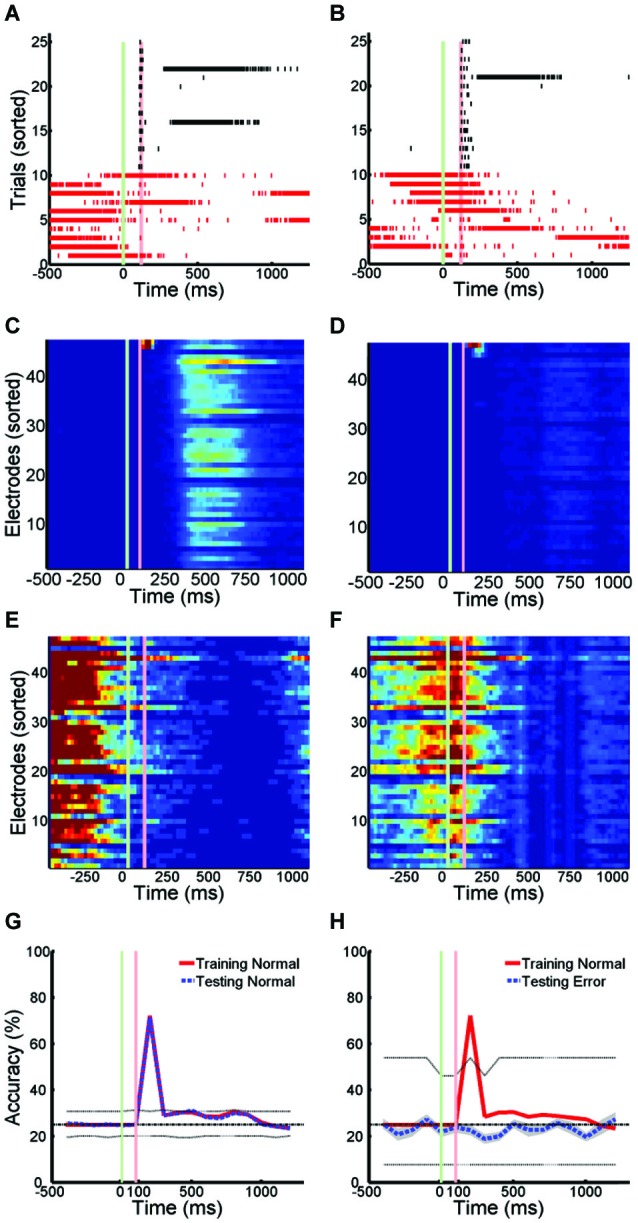
**Active Stimulus Memories are Disrupted by SNBs. (A,B)**: Peristimulus raster plots of network wide responses to the presentation of two random dot stimuli (left and right). Each row represents a different stimulus presentation. Trials are sorted into control (black ticks) and trials with SNBs (red ticks). Green and red vertical lines indicate the onset and offset of stimulus. Time is on the *x*-axis (ms). **(C,D)** Average firing rate in 20 ms bins during trials without SNBs. Colour map range is 0 to 8 Hz. Electrodes sorted by firing rate. **(E,F)** Average firing rate in 20 ms bins during trials with SNBs, color map range 0 to 20 Hz. **(G)** Time series measuring stimulus information during trials without SNBs. Data points computed using SVMs to classify spike counts in 100 ms bins. Four stimuli were presented to network and chance accuracy is 25% (dash-dot line). Solid lines indicate classification accuracy on training (red) and testing (black) trials. Classifier significance computed by taking the best and worst classifications (95th percentile) after random shuffling of target labels (dotted lines). **(H)** Time series of stimulus information during trials with SNBs computed using SVMs trained on control data but tested on trials with SNBs. Other details as **(G)**. All responses from Batch2106-Dish5, DIV8 640 trials (4 stimuli × 160 presentations).

The pattern of activity on trials with SNBs was very different from that seen during control trials without SNBs (Figures 1, 3). During control trials stimuli elicit a reliable spike train (Figures 3A,B, black hash marks) that activates a specific set of electrodes (Figures 3C,D). In trials with SNBs that interrupt presentation of stimuli (Figures 3A,B, red hash marks) can activate very different sets of electrodes (Figures 3E,F). Data is from 640 trials which consist of 160 trials per stimulus (Batch 2106-Dish 5).

When SVMs are trained using data from control trials without SNBs and then tested using previously unencountered data of the same type, the SVMs can classify the unencountered data with a high level of accuracy, usually in excess of 80% (Figure 3G). Overall dishes, the average accuracy of classification in the first 300 ms following stimulus offset was 55.6% ± 25.8% (SD, *n* = 6; ± 0.9% SEM) on training trials and 53.2% ± 25.0% (SD, *n* = 6; ± 0.8% SEM) on testing trials (chance rate of accuracy is 25%). Peak accuracy occurs in the 100 ms time bin 100 ms after stimulus offset: 80.1% ± 18.3% (SD, *n* = 6; ± 0.9% SEM) on training trials and 78.2% ± 16.1% (SD, *n* = 6; ± 0.9% SEM) on testing trials. However, when SVMs that had been trained on control trials are used to classify trials with SNBs, classification accuracy falls to chance levels (Figure 3H). This indicates SNBs destroy stimulus-specific network activity during.

### SNBs use more than one mechanism to disrupt responses to stimuli

**Figure 4:**
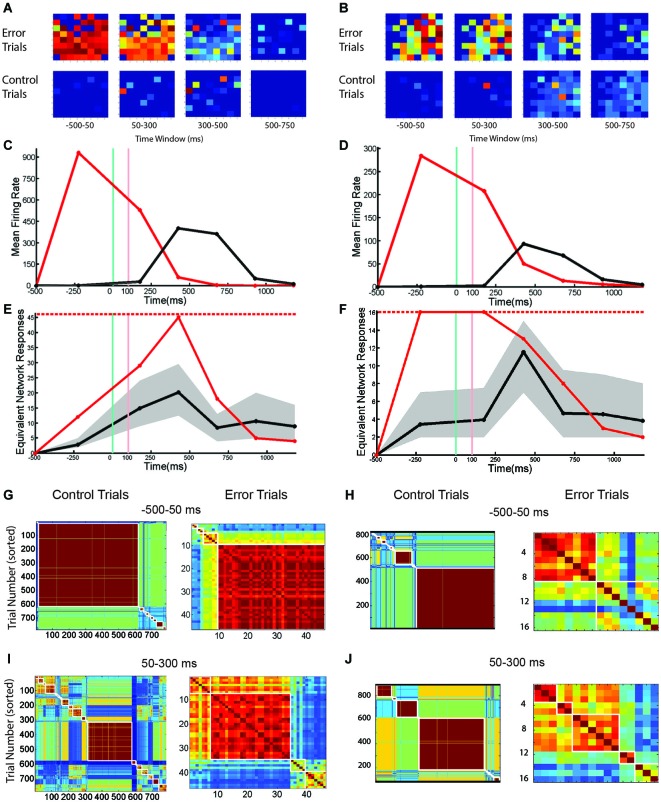
**The Number and Pattern of Units Activated by a SNB is Network Specific. (A,B)** Active units during trials with SNBs (top row) and control (bottom row) for two networks: Batch1905-Dishes 1, DIV9 and 4, DIV11 (left and right). Each 8 × 8 array is laid out in the same configuration as the recording electrodes. Color map indicates probability that a given unit is active in specified time bin. **(C,D)** Mean firing rate (spikes/s) during control (black) or trials with SNBs (red). **(E,F)** Entropy time series. The entropy during trials with SNBs (solid red line) measures how many unique network response patterns were seen in each time bin (*y* = axis counts the equivalent number of unique network response patterns associated with the entropy value, see Materials and Methods). The number of trials with SNBs is the upper limit on entropy (dashed red line; **E**: 46 trials with SNBs, **F**: 16 trials with SNBs). Solid black line indicates the average entropy for 46 control trials (**E**; sampled from 783 control trials, 46 trials with SNBs) or 16 control trials (**F**; sampled from 816 control trials, 16 with SNBs). Trials without SNBs were resampled 300 times and 99th percentiles are shown in gray. Other details as in legend of Figure 1. **(G–J)** Cross-correlation matrices computed at two time points for control (left) and trials with SNBs (right). Trials are sorted to form clusters of similar network responses (red squares). Each cell in the cluster compares the binary response vectors from the two trials as indicated by row and column position and the Color map indicates the correlation distance (response similarity) between the two binary network activity vectors (range = 0 to 1). **(G,H)** Network responses from the first time bin, prior to stimulus presentation, ranging −500 ms to 50 ms post-stimulus. **(I,J)** Network responses from the second time bin, during and after stimulus presentation, ranging from 50 to 300 ms post-stimulus.

Trials with SNBs were analyzed from four cultured neuronal networks (1905- Dish 1, Dish 4; 0504- Dish3; 2106- Dish 3). As described in the Methods section, 12 time bins of data were standardized for each network and correlations computed on the pooled 48 data points. These comparisons reveal that the mean firing rate, number of active channels and normalized entropy are all positively correlated. In particular, mean firing rate was positively correlated with both the normalized entropy and number of active units (*r* = 0.40, *p* = 0.0052; *r* = 0.96, *p* < 0.0001) and the number of active units was positively correlated to the normalized entropy (*r* = 0.48, *p* = 0.0008). These correlations suggest that a simple dynamical model can explain the results: SNBs are associated with the recruitment of additional units, the activation of which increases the mean firing rate and results in higher entropies because more active units mean more unique patterns of network activity. However, when SNB responses were examined on a case by case basis, this trend did not hold for all the networks. Figure 3 displays data from two neuronal networks (Batch 1905, Dishes 1 and 4). The figures in the left column present data from a neuronal network where this correlation does not hold during the occurrence on an SNB (Figures 4A,C,E,G,I). The figures in the right column present data from a second neuronal network where this correlation does hold during SNBs (Figures 4B,D,F,H,J). These contrasting results indicate that the simple mechanism proposed previously does not explain the behavior of SNBs in all networks, warranting closer examination of network responses. In order to understand why different networks are associated with different response patterns, data from trials with and without SNBs were analyzed.

Figures 4A,B show the pattern of activate channels during trials with SNBs (top row) and control trials (bottom row) using the same single-stimulus presentation protocol detailed in Figure 3. These images demonstrate that at the time of stimulus presentation (or SNB occurrence) more units are active during trials with SNBs than control trials. This difference in activation level is also reflected by a large difference in the overall mean firing rate during both trial types (Figures 4C,D). When just these two statistics are considered, the response dynamics of the two networks are qualitatively very similar despite the large differences in the overall mean firing rate, and number of active channels between the two networks (the firing rate in the first network is larger by a factor of 4 and number of channels larger by a factor of 1.5). When entropy is considered, the responses of the networks during control trials continue to be very similar: entropy peaks in the third time bin and then declines (Figures 4E,F, black lines). This indicates that for control trials mean entropy tracks mean firing rate. However when trials with SNBs were considered, very different trends in entropy were observed between the two networks. For the second network, entropy follows the trend outlined previously and increases during an SNB along with mean firing rate and the number of active channels (Figure 4F, red line). Whereas in the first network, entropy actually decouples from the mean firing rate during an SNB (Figure 4E, first and second time bins) and doesn’t peak until the firing rate subsides a bit in the third time bin. Hence the entropies of networks can be significantly different in the time bins where SNBs occur.

To determine whether SNBs activate a single stereotyped pattern, act like white noise, or activate a small number of different stereotyped patterns, the similarity of network responses was assessed using cross correlation and similar responses were clustered and then ordered within each cluster by the stimulus that was presented on the trial. As qualitative differences in network responses were most profound during the first two time bins, a clustering analysis of these responses was done for both trial types (Figures 4G–J). During control trials, network responses during the first time bin are similar and are composed of one or a few stereotyped responses (Figures 4G,H, left; similar responses are grouped into the same red clusters). In both networks the largest cluster of similar responses in the first time bin corresponds to the trivial case where no units are active. This case reflects low baseline activity and the absence of external stimulation in the first time bin. For the first network 76% of trials have a null response (and hence are similar) while in the second network 63% of trials have a null response. This analysis indicates that the low entropy seen on control trials during the first time bin is due to one type of stereotyped response: no response. In the second time bin an external stimulus is applied to the networks and a number of very different network responses are observed. Here network responses are influenced by the identity of the stimulus that is presented on each trial. Although only four stimuli are presented in nearly equal proportion, many more response clusters are seen, indicating the same stimulus does not always elicit the same response (Figures 4I,J, left arrays). In addition, different stimuli do not always yield different responses—when averaged across both networks, the typical cluster of similar network responses is composed of network responses to about 2 different stimuli (0.9 bits or 1.87 stimuli per cluster). This number is influenced by the algorithm employed and in our hands SVM response classification outperformed all such clustering algorithms.

SNBs occur mainly in the first and second time bins during trials with SNBs. For both networks the largest clusters tended to be in the first time bin, indicating that SNBs are more stereotypical in the first time bin (Figures 4G,H, right). In the first network, for trials with SNBs, 78% of responses during the first time bin are grouped into a single cluster (Figure 4G, right). This cluster was not stimulus-specific and includes network responses to all four stimuli (3.68 stimuli or 1.844 bits). All four stimuli were not equally represented in the cluster because one stimulus is under-represented during trials with SNBs. The remaining 22% of trials form several small clusters. These results suggest that in the first network SNB responses are primarily slightly noisy versions of a single stereotyped response. Clusters in the second network were less well defined. One similarity cluster was composed of about half the trials with SNBs while the remaining trials are fairly unique (Figure 4H). This observation suggests again that most responses are composed of a few stereotyped responses. Analysis of the second time bin in trials with SNBs indicated that network responses tend to group into similar responses that are not sensitive to the identity of the four different stimuli that were presented. For the first network, a single large cluster of trials with similar SNB responses can still be observed (Figure 4I, right). For the second network, the clusters are less similar to one another (Figure 4J, right).

Results from Figure 4 indicate that in the first network SNBs overwrite stimulus information by activating a single noisy stereotyped response, while in the second network there are a couple of noisy stereotyped SNB responses and a number of trial-unique SNB-associated network response patterns.

### SNBs disrupt encoding, storage, and retrieval of stimulus-specific information during a modified paired pulse task

**Figure 5:**
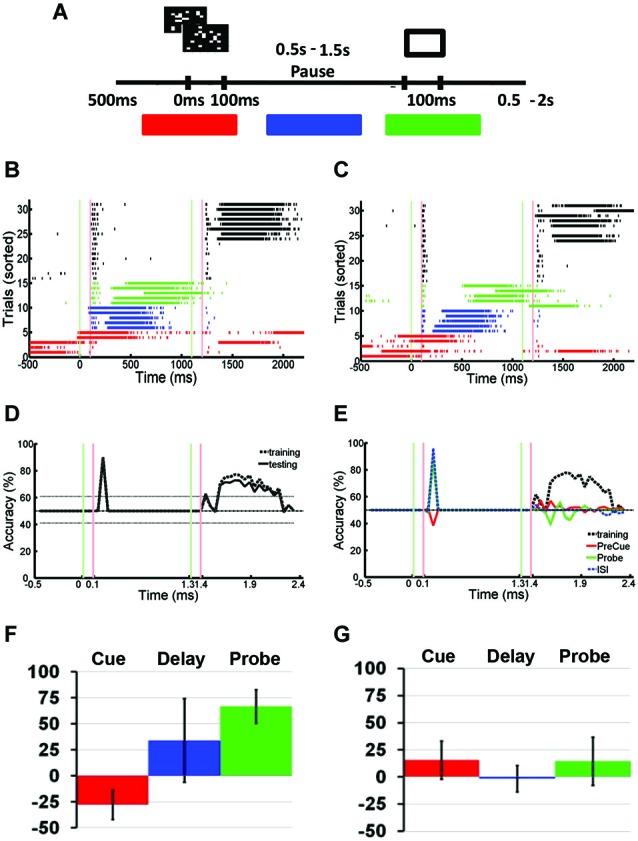
**Spontaneous Network Bursts Disrupts Stimulus-specific Information Stored Using Hidden Memory Mechanisms. (A)** Modified paired pulse task: the sequential stimulus task. Two stimuli were presented sequentially to a neuronal network: a cue stimulus followed by a probe stimulus after a short delay. The cue stimulus could be one of two random dot stimuli. The probe stimulus was fixed for every trial. Marked in red, blue, and green are the cue, delay, and probe phases that define the three different kinds of trials with SNBs. **(B,C)** Peristimulus raster plot of responses to two different stimuli, recorded from a DIV11 neuronal network during the sequential stimulus task. Each row represents a different trial, and trials are sorted into control and trials with SNBs. On trials without SNBs, tick marks are black. Trials with SNBs are colored depending on whether a spontaneous burst was observed during the cue (red), delay (blue), or probe (green) phases. Other conventions as in Figure 3A. **(D)** Time series identifying the amount of cue-related stimulus information across the trial. Time series are constructed as discussed in Figure 3D. SVMs were trained and tested on trials without SNBs. Accuracy of SVM on classifying training trials is shown with dashed lines. Accuracy of SVM classification on testing trials is shown by black solid line. Chance classification is 50%, other conventions as in Figure 3D. **(E)** Time series identifying the amount of cue-related stimulus information on trials with SNBs. The SVM is trained using data from trials without SNBs (dashed black line) and then tested on cue, delay, or probe phase trials with SNBs (red, green, or blue lines, respectively). Other conventions as in **(D)**. Data (A)-(E) from 1905-Dish4, DIV11. **(F)** Mean change in classification accuracy measured during presentation of the cue stimulus for each of the three trials with SNBs (cue phase coded red, delay phase coded blue, probe phase coded green). Change in accuracy characterized as a percent of the classification accuracy during trials without SNBs. Vertical black lines on each bar indicate SEM (*n* = 3, 1905-Dish 4 DIV11, 2106-Dishes 3 DIV10 and 5 DIV9). **(G)** Mean change in classification accuracy during presentation of the probe stimulus. Other conventions as in **(E)**, *n* = 3, 1905-Dishes 4 DIV11, 2106-Dishes 3 DIV10 and 5 DIV9).

A modified paired pulse task was performed to test whether SNBs can disrupt stimulus information stored across delays where no neural activity is measured. The persistence of stimulus-specific information is measured by the adaptation of the network response to the presentation of the second of two stimuli. However, unlike paired pulse tasks, the identity of the first stimulus differs from trial to trial and the experiment aims to uncover whether stimulus-specific information (not simply evidence of past stimulation) is disrupted by SNBs. The task is divided into cue, delay and probe phases (Figure 5A). During the cue phase, one of two possible stimuli is presented. A delay ensues during which no stimuli are presented, followed by the presentation of a single probe stimulus. The response of the network to the probe stimulus is analyzed using SVMs for evidence of cue-dependent adaptation. Figures 5B,C show the responses of one cultured neuronal network to the two different cue-probe sequences shown in Figure 5A. Trials without SNBs were defined as those in which no network bursts occurred prior to presentation of the probe stimulus (Figures 5B,C; black rasters). During these trials the cue changes how the network responds to the probe. When SVMs were trained to distinguish network responses to either the cue or the probe, they were able to accurately determine which stimulus had been presented during the cue phase of the task on 71.1% ± 4.3% of trials without SNBs SEM, *n* = 3; Figure 5D). SVMs were capable of classifying the adapted responses of the network during the probe phase equally well (72.6% ± 6.4%, SEM, trials without SNBs, *n* = 3).

In order to compare this data with previous IED experiments, trials with SNBs were segregated into three classes depending on whether the SNB occurred prior to cue onset (Figures 5B,C; red rasters), during the delay (blue rasters), or during the probe presentation (green rasters). SVMs could not accurately classify network responses to the probe for any of the three classes of trials with SNBs. This was true when SVMs were trained using trials without SNBs (Figure 5E) or trials with SNBs. However, different results were seen among each of three classes of SNB-containing trials when these SVMs were tested on their ability to correctly distinguish cue stimuli. On delay or probe phase trials with SNBs (Figure 5E; blue and green lines), SVMs were able to correctly classify network responses to the cue stimulus. However, on cue phase trials with SNBs, SVMs failed to correctly classify the cue (Figure 5E; red line).

Figures 5F,G summarize the results of three experiments, presenting the average accuracy that SVMs trained using control trials were able to classify SNB-trial network responses to cue stimuli (Figure 5F) and probe stimuli (Figure 5G). All three types of trials with SNBs result in diminished capacity for SVMs to classify network responses to the probe stimulus (Figure 5G). As expected, SVMs were unable to classify network responses to the cue stimulus during cue phase trials with SNBs (Figure 5F; red bar) but were able to classify probe phase trials with SNBs (Figure 5F; green bar). In the case of delay phase trials with SNBs, classification results varied across dishes. An analysis of seven dishes found that this variability correlated with the delay between the cue-elicited response and the onset of the network burst. When there was a long lag between the 100–200 ms cue-elicited response and the onset of a network burst, SVM classifiers that were trained on control trials generalized well to delay phase trials with SNBs. In cases where the network bursts followed quickly after the initial cue-elicited response, classifiers generalized poorly. As a result there is a large standard error for the blue bar in Figure 5F.

### Network excitability determines the pattern of network activity

**Figure 6:**
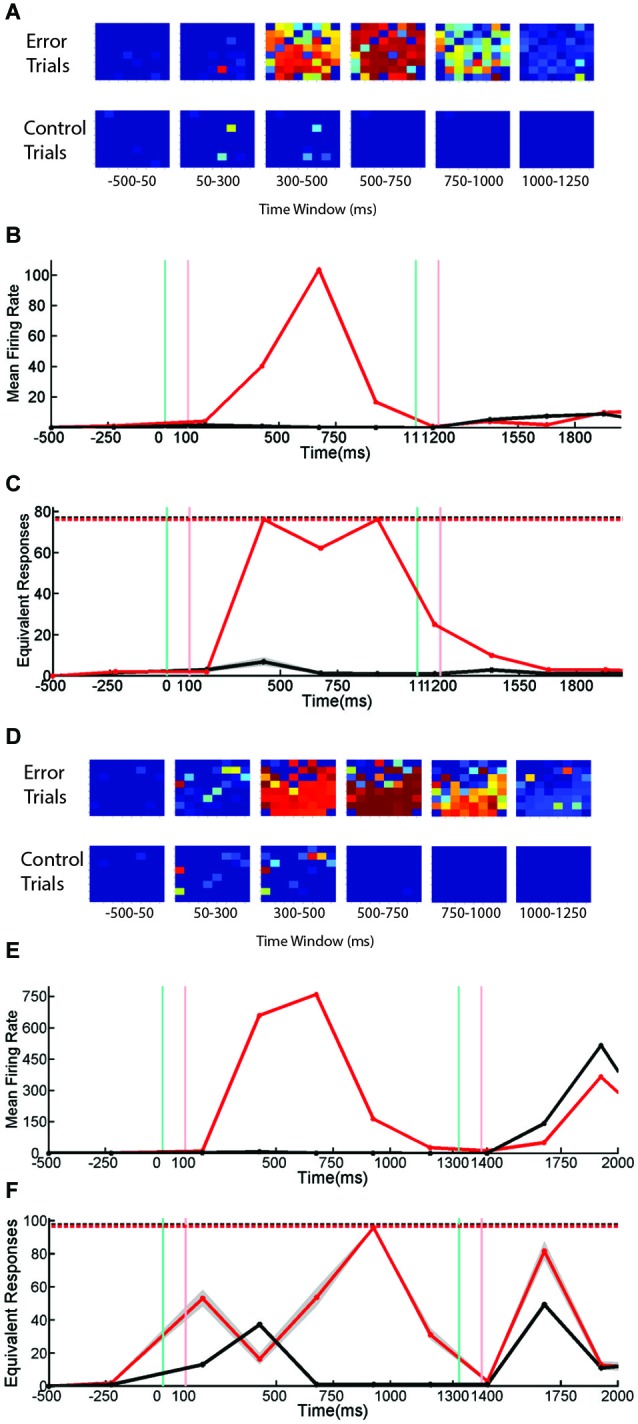
**Firing Rate, Active Channels, and Entropy of SNBs During Sequential Stimulus Trials**. Data from sequential stimulus trials are presented as time series. **(A)** 8 × 8 arrays, indicating probability that individual units are active from neuronal network, 1905-Dish4 DIV11. Each array corresponds to one time bin. Conventions as in Figure 3A. Trials without SNBs consist of trials where no SNB occurred. Trials with SNBs consist of trials where an SNB occurred during the delay phase of sequential stimulus task, a modified paired-pulse paradigm. **(B)** Mean firing rate recorded across all units, averaged by trial (spikes per second). Black line indicates trials without SNBs, red line trials with SNBs. Every time bin after the first is 250 ms. **(C)** Entropy of network responses, plotted as the equivalent number of unique network response patterns. 76 trials with SNBs are plotted in red. 76 trials without SNBs (randomly sampled 300 times from 150 trials without SNBs) are plotted in black. Other conventions as in Figure 4C. **(D)** 8 × 8 arrays associated with a second neuronal network, 2106-Dish 5 DIV9. **(E)** Mean firing rate of second network in spikes per second. **(F)** Entropy of second network. 98 trials with SNBs are shown in red. Black line represents the mean entropy of 98 trials without SNBs drawn from a pool of more than 400 trials without SNBs by 300 re-samplings. 99th percentiles of the resampling are shown in gray. Other conventions as in Figure 4C. When data from 3 cultures (1905-Dish 4 DIV11, 2106-Dishes 3 DIV10 and 5 DIV9) are pooled (*n* = 60 observations: 2 trial types across 10 time bins from 3 cultures with scores standardized within each culture), the correlation between the mean firing rate and the normalized entropy was *r* = 0.34 (*p* = 0.008); the correlation between the number of active channels and normalized entropy was *r* = 0.35, (*p* = 0.007); and larger numbers of active channels are correlated with higher firing rates, *r* = 0.92 (*p* < 0.0001).

When network responses across all time bins were analyzed, the same correlations found in the previous task were found in the sequential stimulus task: firing rate, the number of active channels and entropy are all positively correlated. In order to investigate whether SNBs that occur during the delay phase of the task might have properties different from those that interrupt the presentation of cues, delay phase network responses on trials with and without SNBs were collected and analyzed. Inspection of delay phase responses revealed these correlations do not hold in all networks. Figure 6 presents data from two networks that respond differently when stimulus presentation is interrupted by an SNB. Figures 6A,D display the responses of two different cultured neuronal networks during delay phase trials with SNBs (top row) and trials without SNBs (bottom row). When SNBs occur during the delay phase they recruit a large number of units from across the network. In contrast, on trials without SNBs, only a few units are activated by light stimulation or during the delay. However in both cases, increases in mean firing rate track increases in the number of activated units (Figures 6B,E).

In the first network the overall correlation between active units, firing rate, and entropy mostly holds during both control and error trials (Figure 6C). The entropy on control and delay phase trials with SNBs does not really diverge until the third time bin, which is where an SNB occurs on delay phase trials with SNBs. In the fourth and fifth time bins, the very high entropy of the SNB response continued while activity on control trials diminished to zero. Although this network mostly follows the trends expected by the overall correlation, there are some small variations in entropy that run counter to this the trend. During the fourth time bin when both firing rate and the number of active channels are at their peak, a slight decrease in entropy is observed.

For the second network (Figure 6F), the pattern of entropy during SNBs does not follow the trend expected by the overall correlations. During the cue phase, presentation of the stimulus increases entropy, mean firing rate and the number of active units in both control and error trials. As with the first network, the occurrence of a SNB in the third time bin is associated with higher firing rates and more active channels, however entropy decreases to levels below control trials for the same time bin. Hence the response on SNB-trials was more stereotyped than the response on control trials. In the fourth time bins, the pattern of network activity on trials with SNBs became more stochastic and entropy increased. However entropy did not peak until the fifth time bin, which is associated with a markedly lower firing rate and average number of active channels. While activity is lower during control trials, the overall patterns in mean firing rate, active units, and entropy did not deviate from expected overall correlations. Interestingly, in both networks, peak firing rate is associated with a reduction in entropy, and in adjacent time bins where firing rate is lower, entropy is higher.

**Figure 7:**
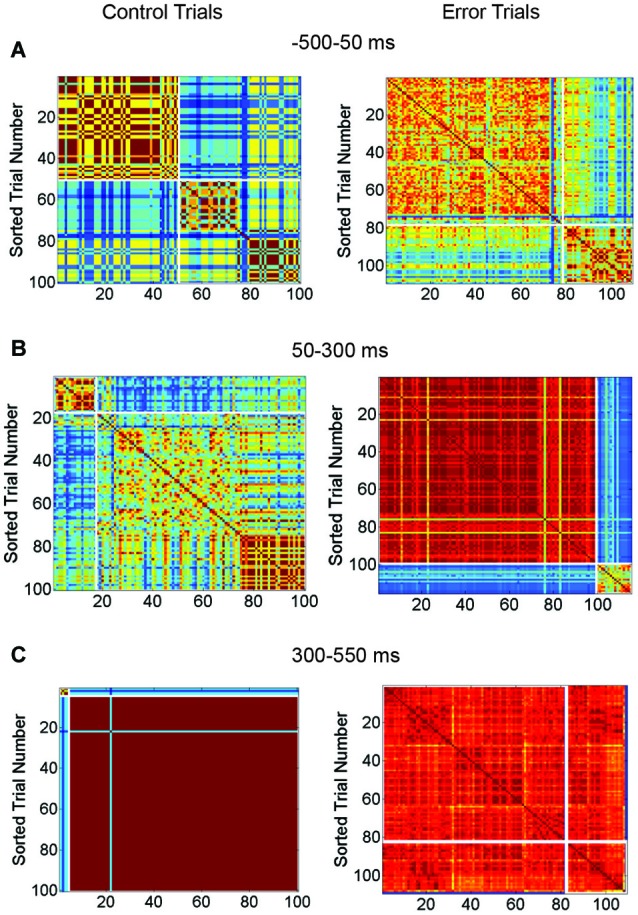
**Matrix Comparing Similarity of Bursting and Non-Bursting Responses During Sequential Stimulus Trials**. Matrices encode the similarity of network responses. Color of cells indicates the similarity of responses by correlation strength. Average entropy of the clusters is given at top of each figure. Other conventions as in Figure 4. **(A)** Clusters of similar network responses during time bin when stimuli are presented (50 ms to 300 ms) for control (left) and trials with SNBs (right). **(B)** Clusters of similar responses during the time bin (300 ms to 550 ms) when the SNB usually occurs on trials with SNBs (right). Data from trials without SNBs also shown (left). **(C)** Clusters of similar responses during the third time bin (550–800 ms) for trials without SNBs the relationship between similar network responses and stimulus identity has deteriorated in this time bin. Trials with SNBs remain unselective for stimuli. (1905-Dish4 DIV11).

Network responses were analyzed and grouped by similarity using cross-correlation matrices (Figure 7). Control trials (from the network shown in Figures 6D–F) are shown at left and trials with SNBs at right. The identity of the stimulus influenced the occurrence of a network burst and as a result, the two stimuli used in these experiments are not equally distributed among control and SNB trials. Control trials are slightly biased towards stimulus 2 (stimulus label distribution entropy = 0.92 bits) and trials with SNBs are biased toward stimulus 1 (stimulus label distribution entropy = 0.77 bits). When all three cases are considered, the average entropy of trials without SNBs was 0.94 bits and trials with SNBs, 0.56 bits (*n* = 3).

In Figure 7A, the response of the network to stimulus presentation is analyzed. During control trials the first cluster of similar network responses was found to be selective for stimulus 2 (0.52 bits) while the second cluster was nonselective (0.99 bits). During trials with SNBs the same pattern was found; the first cluster of similar responses was selective for stimulus 1 (entropy = 0.29 bits) while the second cluster was relatively nonselective (entropy = 0.82 bits). During the next time bin (Figure 7B), the SNB occurred and on trials with SNBs most of the network responses were grouped into a large red cluster that was mildly selective for stimulus 1 (0.7 bits). The second, smaller cluster was nonselective (0.94 bits). During control trials, both clusters of similar network responses mildly favored stimulus 2 (0.64 bits and 0.78 bits). In the next bin (Figure 7C) control trials went silent and were stimulus non-selective (0.93 bits). A remnant of the SNB continued during trials with SNBs and the network responses were, with the exception of one outlier, grouped into one cluster. This cluster was nonselective (0.8 bits).

Although not displayed, the second network (Figures 6A–C) had similar trends: stimuli were unequally distributed amongst trials with and without SNBs so that control and SNB trials had entropy values of 0.98 and 0.49 bits, respectively. During stimulus presentation, similar, highly selective responses were observed for both control and SNB trials (average of 0.14 bits per cluster). During the next time bin, where the SNB occurred, responses on both control and SNB trials were relatively nonselective, with the largest control cluster having an entropy of 0.92 bits and largest cluster of SNB responses having an entropy of 0.22 bits. In the next bin, control trials were silent with an entropy of 0.98 bits and the SNB trials were all clustered into a single SNB response except for 3 outliers. The entropy of that SNB response was 0.37 bits.

**Figure 8:**
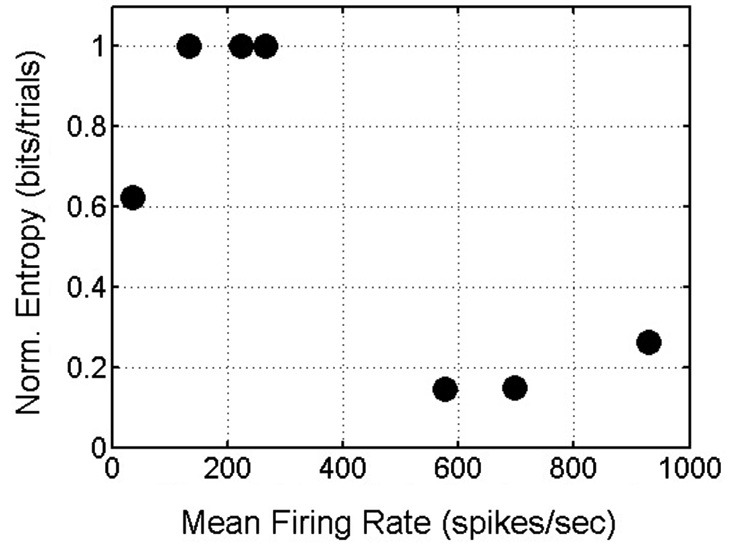
**Scatterplot of Mean Firing Rate and Normalized Entropy**. Data from 7 spontaneous network bursts (SNBs) from 4 networks (1905-Dishes 4 DIV11 and 5 DIV9, 2106-Dishes 3 DIV10 and 5 DIV 9) in 7 experiments (circles). Networks with higher mean firing rates during SNBs have lower normalized entropies; networks with lower mean firing rates during SNBs have higher entropies (*r* = −0.78, *p* = 0.037).

In summary, delay-phase SNBs, like pre-stimulus SNBs, recruit one or possibly a few stereotyped patterns of active units. Additional units get recruited in a stochastic fashion. When the observations from all four networks are pooled, a trend in the entropy on SNBs and mean firing rate in SNBs emerges: networks that have a higher mean firing rate during SNBs (>500 spikes/s) experience a reduction in entropy during SNBs while networks that have a lower mean firing rate during an SNB (<500 spikes/s) experience an increase in entropy during SNBs (Figure 8).

## Discussion

The results from the experiments described above demonstrate that stimulus-specific information can be represented in randomly organized neuronal network formed from disassociated cortical neurons and that this information is disrupted when synchronized bursts of network activity take place. Specifically, when complex optical stimuli are presented to optogenetically modified neuronal networks, different stimuli elicit different patterns of neural activity and these patterns are disrupted by SNBs (Figure 3). SVMs can be trained to recognize which stimulus is being presented on single trials by classifying the pattern (unit number) and energy (firing rate) of neural responses (Figures 3, 5). The activity of many units activity is redundant and in agreement with a previous report, most of the stimulus information can usually be extracted from about 4–5 units or 10% of recorded units (Dranias et al., 2013). The disruption of these “active” representations of stimulus information by SNBs has not been shown previously (Figures 3H, 5E). Cultured neuronal networks can also store stimulus-specific information across delays of several hundred milliseconds where no neural activity has been measured (Dranias et al., 2013). This was revealed using a modified paired pulse experiment in which these “memory traces” are likely represented by the adaptation of neurons or synapses and it is shown here that this stimulus-specific adaptation is disrupted when SNBs occur during stimulus presentations or during the delay (Figures 5B,C,E, 8). Together these findings demonstrate networks bursts disrupt active and hidden stimulus memory.

The structure of SNBs was characterized in addition to measuring the effects of SNBs on stimulus information. A correlation between entropy, firing rate, and the number of active channels was observed. This correlation suggests a simple and intuitive model that describes network dynamics during an SNB: SNBs recruit additional units, increasing the overall firing rate. With more units active and higher firing rates, more distinct patterns of network activity are possible, hence network entropy increases. However, not all the networks seemed to follow this simple model of network dynamics. For some networks increases in firing rates and active units did not increase entropy (Figure 8).

Since the trend in entropy wasn’t constant, a cluster analysis was performed to look at the structure of network responses. It was hypothesized that SNBs either act as a noise source that corrupts the representations of stimuli or that they overwrite stimulus-elicited signals by saturating the network with activity. The results from the cluster analysis were similar for trials with SNBs from the single stimulus and sequential stimulus experiments: SNBs do not act as white noise, nor do they simply saturate all the active units with activity. Instead SNBs tend to activate one or a few stereotyped patterns that are noisy and the number of different stereotyped responses varies between dishes (Figure 8). In some networks, SNB responses are highly reproducible and form almost a single cluster (Figures 4E,G). In other networks, the SNB responses are more diffuse with different patterns (Figures 4F,H). These results seem to occupy a middle ground: some networks have stereotyped bursts while others have several different noisy stereotyped response patterns (Figures 4, 6, 8).

Work by Shew and others (Shew et al., 2009, 2011) provide an explanation for this behavior. These researchers were exploring how the balance between excitation and inhibition can influence the capacity of neuronal networks to represent information. The ability of a network to store information is limited by how many states the network can occupy (Shew et al., 2011). Their experiments demonstrate that an “inverted-U” describes the relationship between network excitability and network entropy. When a network is too excited it saturates and cannot occupy more than one state. When a network is too inhibited, there is no activity and again no information can be represented. This rule is consistent with the observations made here (Figure 8). However, in these experiments no pharmacological agents were applied so the observed differences in entropy reflect the intrinsic excitability of different neuronal networks. Some networks are more excitable (have a higher mean firing rate during SNBs) and in these networks an SNB will quickly saturate all available units, decreasing entropy. Other networks are less excitable and SNBs simply recruit more units, increasing network entropy (Figure 6). Figure 6C demonstrates that this rule is at play even in less excitable networks; when firing rate peaked in this network, entropy actually decreased slightly. In terms of applications to understanding epilepsy and IEDs, the balance of excitation and inhibition in a network is a property known to be critical in epilepsy (Raichman and Ben-Jacob, 2008). Understanding how information representations are changed when pharmacological agents are used to alter the balance of excitation and inhibition in intrinsically excitable networks is an area of future investigation.

The entropy time series provides some additional observational evidence to the generally acknowledged temporal evolution of network responses to stimulation. Specifically it has been noted that there is an initial orderly response to a stimulus that decays into chaotic randomness (Jimbo et al., 2000; Kermany et al., 2010). On trials without SNBs, entropy increases slightly during stimulus presentation and then shows a larger increase just after stimulus presentation when network responses transition into disorder (Figures 4E,F, 6C,F).

The current study leverages a technical advantage to elaborate the findings of previous paired pulse experiments and answers a somewhat more difficult question: does stimulus-specific information survive an SNB? In a sequential stimulus or modified paired-pulse task, a neuronal network will normally respond to the second stimulus with an adapted response whose recruitment and activity levels vary depending on the identity of the first stimulus (Figure 5D). SNBs disrupted any dependency of the response of the second stimulus on the identity of the first stimulus (Figure 5E). When an SNB occurs during cue presentation, cue-specific information is not encoded into network responses, and no stimulus-specific adaptation of network responses to the probe stimulus is detected. When an SNB interrupts presentation of the probe stimulus, no cue-specific information can be found in the network response to the probe, though the network response to the cue remains intact. Finally, when an SNB occurs during the delay phase, the network response to the probe no longer reflect stimulus specific information (Figure 5G).

More general application of these results requires clarification of what the behavior of this *in vitro* model of an isolated network of cortical neurons has in common with the behavior of networks *in vivo*, which are an integral part of a functional brain. One property both networks appear to have in common is the ability to represent different stimuli using spatiotemporal patterns of activity in neural circuits (Buonomano and Maass, 2009). In cultured neuronal networks, different electrical stimuli can be differentiated by the paths or circuits of neurons they activate (Shahaf et al., 2008). This observation is confirmed in our studies as the SVMs we use to identify stimuli act by distinguishing stimuli on the basis of which units are recruited and their firing rates (spatial pattern and energy). The heat maps in Figures 3C,D also show that different patterns of activation can be associated with different stimuli. The ability of SNBs to recruit additional units and synchronize their activity provides an explanation for how they are able to devastate stimulus representations: SNBs recruit units from across isolated parts of the network and provide these units with synchronized input. The response elicited by an SNB is usually longer in duration and higher in energy than optogenetic stimulation so it is natural for the stimulus-specific pattern of adaptation induced by optogenetic stimulation to be disrupted and SNB. The ability of a network to store stimulus information using different spatial patterns of activity, of networks to process different stimuli in stimulus-specific circuits, of neurons to maintain traces of past activation neural activity, and of network bursts to recruit neurons and synchronize activity are all related to fundamental network mechanisms shared by networks *in vitro* and *in vivo*. Because of these shared properties and the relative difficulty of using microelectrodes and making unit recordings *in vivo*, this study provides observations on how SNBs destroy stimulus information that can serve as a guide for future hypotheses regarding cortical tissue that is epileptogenic and prone to IEDs. It will be interesting to see if IEDs in a cortical network have properties different from those that would be expected from a generic neural network formed from dissociated cortical neurons. If so, these results might help to reveal those principles.

Returning to the question raised in the original study by Kleen et al. (2010) that motivated this investigation: whether bursts of epileptiform activity always destroy stimulus information stored in an isolated neuronal network. The results of the present experiments, grounded in more basic processes and using microelectrodes and unit recordings, indicate that SNBs do indeed destroy stimulus specific information, regardless of timing. However there are a few questions and avenues of investigation left unanswered. First it appears that while SNBs destroy stimulus-specific information, these bursts have a nontrivial entropy and may convey some information. One piece of information that survives an SNB appears to be nonspecific information about stimulation. Further analysis of this question might be an interesting avenue of future investigation. Another question that this research didn’t examine but might be relevant to more general questions is whether isolated neuronal networks are capable of representing information about more than one stimulus simultaneously. This question appears to relate to the ability of a network to harbor isolated representations of stimuli.

Relating to statistical methods, the performance of the classifier on training data was cross-validated with testing data not encountered during classifier training using the method known as repeated random subsampling (where data is repeatedly randomly partitioned into training and testing sets). The classifier was trained and tested 50 times with different random samples of data. The average accuracy or correct classification rate provides a measure of classifier reliability, similar to how its complement, the misclassification rate, might be used in other papers (or derivative measures that compute a loss function based on the misclassification rate, e.g., kfoldloss). Our approach is approximately equivalent to a 4-fold cross validation (or k-fold validation where *k* = 4, which divides data into 75% training and 25% testing), but instead of cycling through the 4 folds, we test and train on 50 different random samplings. K-fold cross validation has the disadvantage that it generally needs larger data sets (i.e., the number of trials divided by k (size of the fold) should be large enough the fold is likely to be a fair sample. The data sets used here typically have 100–500 trials per class so it is unlikely that a single k value larger than 4 could be selected to analyze all data sets. Hence a k-fold cross validation approach is unlikely to produce results different than those observed using random sub-sampling.

IEDs are difficult to study *in vivo* and there have been no experiments done to establish their impact at the neuronal circuit level. This study provides observations on how SNBs destroy stimulus information that can guide future hypotheses. The aim of this study was to provide insight into the kinds of neural dynamics that explain how synchronized bursts of neural activity can disrupt cognitive processing. Because of advances in stem cell technology, the development of new *in vitro* models of basic processes relevant to cognitive and neurological disorders has become increasingly relevant (Chiappalone et al., 2003; Berger et al., 2011; Durnaoglu et al., 2011; Hales et al., 2012; Stephens et al., 2012). The ability to culture human neurons derived from patients with neurological diseases and to test those cells using *in vitro* drug protocols will help researchers develop individualized treatments for patients and perhaps even aid in the development of new drugs for controlling negative symptoms.

## Conflict of interest statement

The authors declare that the research was conducted in the absence of any commercial or financial relationships that could be construed as a potential conflict of interest.

## Footnotes

^1^www.brainbitsllc.com

^2^www.neuroshare.org
